# Optimization of Patient Management in the Gynecology Emergency Department Using Point-of-Care Beta hCG

**DOI:** 10.3390/diagnostics12071670

**Published:** 2022-07-09

**Authors:** Mehdi Brousse, Anne-Sophie Bargnoux, Caroline Courtais-Coulon, Stéphanie Badiou, Nils Kuster, Clara Compan, Florent Fuchs, Jean-Paul Cristol

**Affiliations:** 1 Département de Biochimie et Hormonologie, CHU de Montpellier, Université de Montpellier, 34295 Montpellier, France; m-brousse@chu-montpellier.fr (M.B.); as-bargnoux@chu-montpellier.fr (A.-S.B.); caroline-coulon@chu-montpellier.fr (C.C.-C.); s-badiou@chu-montpellier.fr (S.B.); n-kuster@chu-montpellier.fr (N.K.); 2 Département de Biochimie et Hormonologie, PhyMedExp, INSERM, CNRS, CHU de Montpellier, Université de Montpellier, 34295 Montpellier, France; 3 Département de Gynécologie-Obstétrique, CHU de Montpellier, Université de Montpellier, 34295 Montpellier, France; c-compan@chu-montpellier.fr (C.C.); f-fuchs@chu-montpellier.fr (F.F.); 4 Inserm, CESP Center for Research in Epidemiology and Population Health, U1018, Reproduction and Child Development, 94807 Villejuif, France; 5 Desbret Institute of Epidemiology and Public Health, Université de Montpellier, 34093 Montpellier, France

**Keywords:** POCT, βhCG, length of stay, emergency department

## Abstract

Background: Point-of-care testing (POCT) provides shorter turn-around times and, in many cases, potentially improves medical decision making. The AQT90 FLEX^®^ benchtop immunoanalyzer (Radiometer Medical ApS, Copenhagen, Denmark) allows for the determination of beta-human chorionic gonadotropin (βhCG) in 18 min. The main aim of this study was to evaluate the impact of measuring βhCG using the AQT90 analyzer in the gynecology emergency department (ED) compared to the standard practice of using central laboratory blood testing on the patient length of stay (LOS). Methods: The evaluation consisted of two parts. The first one, conducted in the central laboratory, focused on the analytical performances of the AQT βhCG assay. The second one, conducted in the ED, aimed at determining the impact of POCT βhCG implementation on the timeframe in which ED patients require βhCG assessment. Results: The within-lab imprecisions at the mean values of 17 and 287 IU/L were 2.7% and 3.7%, respectively. Using Deming regression (*n* = 60), the following equation was obtained in the central lab: AQT90 βhCG = 1.1 Roche βhCG—12.9 (r = 0.997). The implementation of POCT βhCG in the ED significantly reduced patient LOS (145 (90–212) min vs. 205 (155–265) with and without AQT90, respectively, *p* < 0.001). At the 2 IU/L decision level, a 99.7% agreement with the Roche assay was reported (kappa statistics, 0.99). Conclusions: We confirm that the analytical qualities of the AQT 90 were in line with those obtained in the central lab. The implementation of the POCT βhCG is associated with a shorter LOS in the ED due to the faster availability of the results and the faster decision-making possibilities.

## 1. Introduction

Overcrowding in emergency departments (ED) is a widely recognized problem that negatively impacts patient care [1,2]. Given that up to 70% of medical decisions are based on laboratory testing [3], faster turn-around times (TAT) with point-of-care testing (POCT) appears to be a promising way to improve both clinical decision making and clinical effectiveness [4,5]. However, better patient outcomes [6,7] can only be achieved if the POC diagnostic is performed by a trained healthcare professional and if the results obtained are accurate and reliable [8]. In the gynecology ED, pregnancy complications require a rapid and accurate diagnostic [9]. In this context, the TAT of beta-human chorionic gonadotropin (βhCG) result is essential, but limited published data describe the effect of POCT βhCG on ED diagnostics and logistics [10,11]. Legoupil et al. [10] evaluated the performance of a qualitative screening test for βhCG in the whole blood of women consulting in early pregnancy units for vaginal bleeding and/or pelvic pain. Although POCT βhCG reduced the waiting time to the result by approximately 85 min, this study did not assess the improvement in the time spent in early pregnancy units. Moreover, the βhCG qualitative tests performed with POC devices are susceptible to false negative results at low concentrations, and the sensitivity is insufficient to detect early pregnancy [12,13,14]. To the best of our knowledge, no study has addressed the impact of quantitative hCG measurement in gynecology EDs. Three POCT quantitative hCG assays performed in whole blood are commercially available in France: the Radiometer AQT90 FLEX βhCG test kit [15], the Siemens Stratus CS Acute Care βHCG assay [16] and the Abbott i-STAT Total βhCG) assay [17]. These POCTs provide βhCG results in 10 to 18 min [15,16,17].

Therefore, the main objective of this study was to evaluate the impact of the implementation of the AQT90 FLEX^®^ immunoanalyzer (Radiometer Medical ApS, Copenhagen, Denmark) for βhCG determination in the gynecology ED on the patient LOS. The secondary objectives were to assess the imprecision of AQT90 βhCG, to compare βhCG measurement between AQT90 and the central laboratory standard and to evaluate the delay between the patient arrival in the ED and the reporting of the results before and after the implementation.

## 2. Materials and Methods

### 2.1. Study Design

The study was conducted in the gynecology ED of the Montpellier University hospital (France). All patients arriving in the ED and requiring βhCG evaluation were enrolled. This was a single prospective center with two periods: (1) Period 1—before the implementation of the AQT (from 1 December 2018 to 18 February 2019), when βhCG testing and the medical decision were based on the core central laboratory results; (2) Period 2—after the implementation of the AQT (from 19 February 2019 to 7 April 2019), when the patient management decisions were based on the POCT βhCG results performed by a certified nurse or midwife. This study has been approved by the Institutional Review Board of the Montpellier University Hospital (number 2018_IRB_MTP_10–05).

### 2.2. Study Protocol

The biochemistry central laboratory checked the analytical performances of the Radiometer AQT90 FLEX βhCG assay before implementation. Operators (*n* = 32), mainly midwives, have been trained on this new system. The POC system was connected via the middleware Aqure (Radiometer) to our Laboratory Information Management System, assuring the traceability of the results. In addition, the βhCG test on AQT was integrated in the quality testing procedure of our laboratory, and the internal quality control materials were run and examined by a biochemistry technician.

### 2.3. Verification of the AQT90 FLEX βhCG Analytical Performances

The βhCG immunoassay on the fully automatic continuous-access AQT90^®^ analyzer is a one-step sandwich immunoassay utilizing time-resolved fluorometric detection [18]. The reportable range of the assay is 2–5000 IU/L (defined by the lower detection limit and the maximum of the master curve). Values below the lower detection limit and above the measuring range are reported as <2 and >5000 IU/L, respectively. The 95th percentile βhCG reference value for the AQT90 FLEX^®^ was claimed by the manufacturer as <2 IU/L for pre-menopausal women (*n* = 87) [18].

Imprecision was assessed using the clinical and laboratory standard institute (CLSI) EP15-A3 protocol (one run per day, five replicates per run for five days, *n* = 25 replicates per sample) [19] by the repeated analysis of two levels of the Radiometer liquid immunoassay controls (LQC hCG-CHECK level 1 and 2). To verify the lower limit of quantification (LLOQ), a precision profile was established using a panel of four whole blood pools with target concentrations at approximately 3, 8, 11 and 94 IU/L. The samples were measured 15 times in one run.

The AQT results were compared with those obtained in the central laboratory using the Roche Elecsys hCG + β assay on an e602/cobas 8000 analyzer, an electrochemiluminescent assay calibrated based on the sandwich principle. The reportable range of the assay is 0.100–10,000 IU/L (defined by the lower detection limit and the maximum of the master curve). Values above the measuring range are reported up to 1,000,000 IU/L for 100-fold diluted samples. In the central lab, the decision level for pregnancy is set at ≥2 IU/L, which corresponds to 98.9% of the values obtained from 181 healthy, non-pregnant and premenopausal women (expected values claimed by the manufacturer, study number BO1P019 [20]). Excess whole blood samples were selected and assessed on both AQT and, after centrifugation, cobas 8000 (Roche Diagnostics GmbH, Mannheim, Germany) during a minimum of 5 days to include day-to-day variation.

### 2.4. Endpoints

The primary outcome was the patients’ LOS, computed from admission to the ED until hospitalization/discharge from the ED. The secondary outcome was the interval from blood sampling to the reporting of the results (lab results in phase 1 and POCT results in phase 2). In addition, during phase 2, after measurement on the AQT, the remaining whole blood sample of each patient was sent to the biochemistry central laboratory according to the usual procedures, which allowed us to monitor the comparison and concordance of the results with the Roche assay.

### 2.5. Statistical Analysis

A one-way analysis of variance (ANOVA) was performed for each aqueous control concentration to estimate repeatability (%CV_R_) and within-lab imprecision (%CV_WL_) after carrying out a Grubbs’ test to detect potential outliers [19]. LLOQ was determined by plotting the coefficients of variation (CVs) calculated at each concentration against the mean concentration. A precision profile was established by defining the LLOQ as being the smallest measurable value with an imprecision < 10 and 20%. A Deming regression analysis was performed to compare the data from the different methods. In addition, the scatter of differences was visualized according to the Bland–Altman representation. The mean and limits of agreement, defined as the mean ± 1.96 standard deviation (SD), were computed [21]. The agreement at the medical decision level was calculated using Cohen’s κ-test. The Mann–Whitney test was used to compare the time data before and after the implementation of the AQT. Statistical analysis was carried out with the XLSTAT^®^ software for Windows, version 2016.06.35661 (New York, NY, USA), and the statistical significance was set at *p* < 0.05.

## 3. Results

A total of 1004 patients were included in this study (median age = 28.6 (23.8–24.5) years: 610 women during the 11 weeks before and 394 women during the 7 weeks after AQT implementation in the Gynecology ED). Almost all of the patients (98.9%) went to the ED on their own. The reasons for medical consultation were pelvic or abdominal pain in 35% of cases and vaginal bleeding in 29% of cases. Serial quantitative hCG measurements in the context of pregnancy of an unknown location represented 30% of the cases. The majority of patients (97.8%) were discharged.

### 3.1. Verification of the AQT90 FLEX βhCG Performance

**Figure 1:**
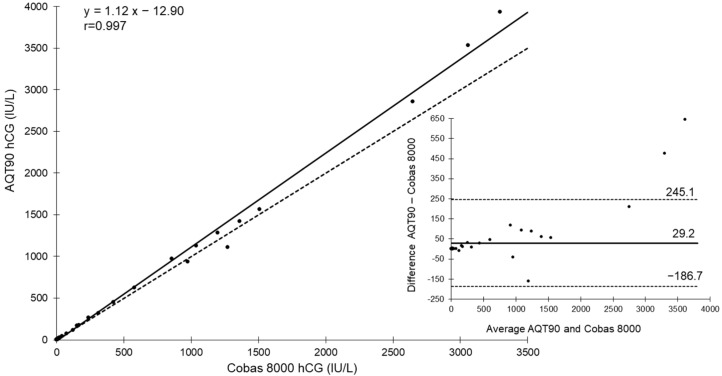
Deming regression (*n* = 60) of hCG on e602/Cobas 8000 (plasma) and AQT90 (whole blood) in the central lab, and Bland–Altman plot of differences against means for patient samples with both analyzers. The Deming graph shows the regression line (solid line) and identity line (x = y, dashed line). For the Bland–Altman representation, the mean (solid horizontal line) and limits of agreement (dashed lines) of the bias (IU/L) were computed.

At the mean value of 17 IU/L (LQC hCG-CHECK level 1), CV_R_ and CV_WL_ were 2.4% and 2.7%, respectively. At the mean value of 287 IU/L (LQC hCG-CHECK level 2), CV_R_ and CV_WL_ were 3.0% and 3.7%, respectively. The LLOQ of the assay was found at 4 and <2 IU/L using CV 10 and 20%, respectively. For the comparison study, the excess whole blood from 60 patients was selected to cover the analytical measuring range of 2 to 5000 IU/L. The following linear regression equation (y = ax + b) was obtained: AQT90 hCG = 1.1 Roche hCG—12.9 (r = 0.997). The mean and limits of agreement (mean ± 1.96 standard deviation) of the difference according to the Bland–Altman’s study [21] between hCG on e602/cobas 8000 and AQT90 were 29.2 ± 215.9 IU/L (Figure 1).

### 3.2. Evaluation of POCT Implementation in the Emergency Department

**Figure 2:**
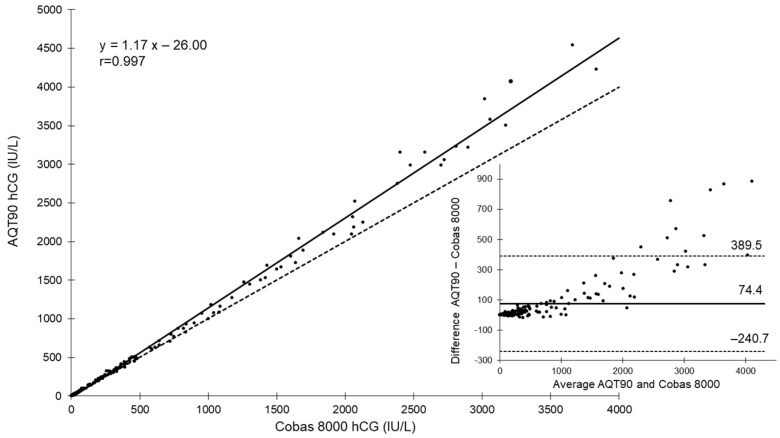
Deming regression (*n* = 176) of hCG on e602/Cobas 8000 (plasma) and AQT90 (whole blood) in the ED, and Bland–Altman plot of differences against means for patient samples with both analyzers. The Deming graph shows the regression line (solid line) and identity line (x = y, dashed line).For the Bland–Altman representation, the mean (solid horizontal line) and limits of agreement (dashed lines) of the bias (IU/L) were computed.

During the 7 weeks of the second phase, samples from 314 patients were assessed on the AQT90 in the ED and then analyzed on the e602/cobas8000 in the central lab. The Deming regression analysis of all samples, except those lower than the LoD (*n* = 123) or higher than the measuring range (*n* = 23), reported a slope of 1.17 and a y-intercept of −26 (Figure 2). The mean bias between the Roche and AQT tests was 74.4 ± 315.1 IU/L. After considering the Roche assay as the comparative method, the overall agreement between both assays was 99.7 at the decision level of 2 IU/L (kappa statistics, 0.99; 95% CI, 0.98–1.00). The only discordant result pair on the 314 samples corresponded to a βhCG < 2 IU/L using the Roche Elecsys βhCG and was equal to 2 IU/L using the AQT βhCG. The within-lab imprecisions generated from our internal quality were 3.9% and 4.6% at the mean values of 17 IU/L and 291 IU/L, respectively.

**Figure 3:**
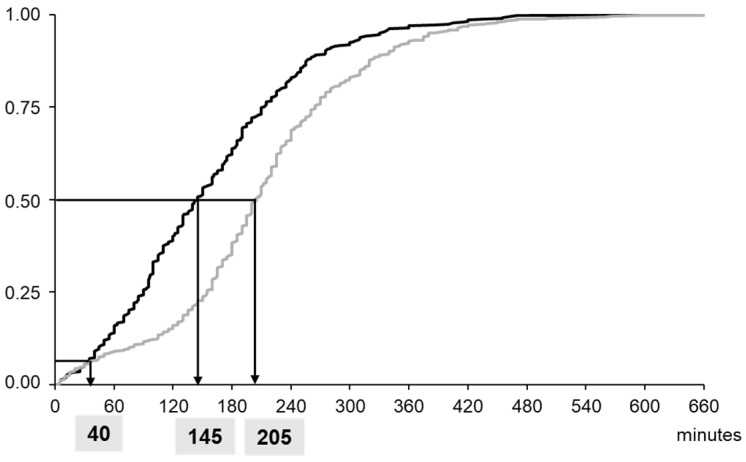
Cumulative frequency distribution of patient length of stay before (grey line) and after (black line) the implementation of AQT90 in the emergency department.

**Figure 4:**
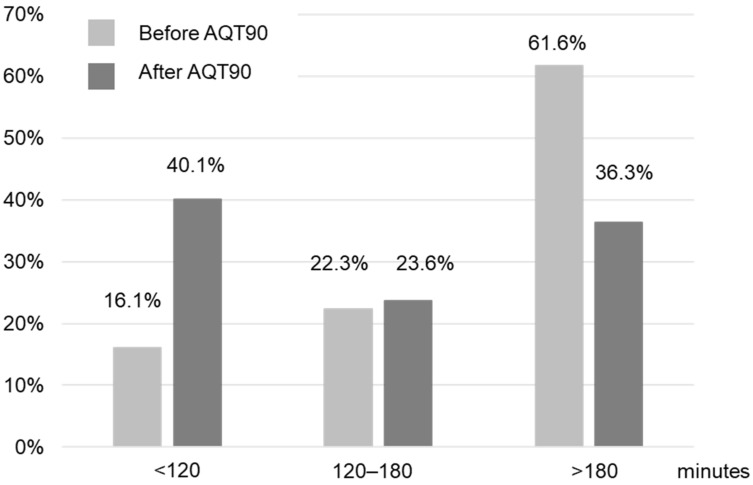
Frequency distribution of the patient length of stay per time period before and after the implementation of AQT90 in the emergency department.

The implementation of POCT βhCG in the ED of gynecology significantly reduced the LOS of the patients from 205 (155–265) (median (interquartile range)) min to 145 (90–212) min for the periods without and with AQT90, respectively (*p* < 0.0001) (Figure 3). The number of patients treated in less than 2 h was increased by a factor of 2.5 (Figure 4). During Phase 1, the central lab hCG results were available within 141 (115–171) min. During Phase 2, the median time taken to obtain the hCG result with POCT after patient sampling was 22 (19–25) min (*p* < 0.001, versus the data for the central lab result).

## 4. Discussion

After the validation of the analytical performances in our central laboratory, the AQT90 POC device for βhCG measurement was implemented in the gynecology ED. This analyzer presents analytical qualities in line with those obtained in the central lab, and the results of this prospective observational study further show a significant reduction in the time spent in the ED.

### 4.1. Analytical Performances

The performances and diagnostic accuracy of the assay were found to be clinically acceptable. The within-lab imprecision was less than 4% and comparable to previously reported CVs of 3.5–3.9% and 2.8–5.1% for level 1 and level 2 QC material, respectively [15]. The present study confirmed a positive bias evaluated at 10% compared to the Roche Elecsys hCG + β method on a e602/cobas 8000 analyzer [15]. The differences observed have been largely attributed to the lack of harmonization of the assays, including the detection of hCG variants, the principle of detection and the calibration process [9,22,23]. Both immunoassays are traceable to the WHO 4th International Standard [24]. Detection on the AQT90 is time-resolved fluorescence using a europium chelate-labelled signal antibody [18], whereas detection on the e602/cobas 8000 is chemiluminescence using a ruthenium chelate-labelled signal antibody [20]. During pregnancy, hCG immunoreactivity depends on both the intact heterodimeric hCG comprising both the α and β subunits and the hCG variants that differ in the protein structure and the carbohydrate content [9,23]. hCG variants consist of free hCG subunits (α or β), degradation products of hCG and glycosylated forms of hCG. There are six major hCG variants: free α subunit (hCGα), free β subunit (hCGβ), nicked (hCGn), nicked free β subunit (hCGβn), β core fragment (hCGβcf) and hyperglycosylated (hCGh). Intact hCG, hCGα, hCGβ, hCGn, hCGβn and hCGh are detectable in blood and urine, whereas hCGβcf is only detectable in urine. Therefore, the analytical specificity for hCG variants depends on the epitopes recognized by the assay antibodies [25]. The Elecsys hCG + β test is known to appropriately detect intact hCG, hyperglycosylated hCG, nicked hyperglycosylated hCG, the βhCG core fragment and the free βhCG subunit [22,23,25]. To the best of our knowledge, no data on the detection of hCG variants by the Radiometer AQT90 FLEX βhCG assay have been previously published. The notice mentions that both intact hCG and free β subunits are recognized by the antibodies used in the βhCG assay. This evaluation is of particular importance since the medical staff should be made aware of the lack of transferability of the hCG results between the central lab and the POCT for patient monitoring with serial measurements. Nevertheless, at the βhCG cutoff of 2 IU/L, neither false positive nor false negative results were observed with this new technology. The only discordant result pair was a βhCG < 2 IU/L using the Roche Elecsys βhCG and was equal to 2 IU/L using the AQT βhCG. The agreement of the AQT90 with the Roche assay using 5 IU/L, corresponding to the level of the medical decision generally used to exclude a diagnosis of pregnancy in premenopausal women [26,27], was 99%, much better than that previously reported at 75% [15]. βhCG qualitative tests on POC devices are susceptible to false negative results because of a lower limit of detection of 10 to 25 IU/L [12,13,14]. The reported LLOQ of 4 IU/L and <2 IU/L, using CV 10% and 20%, respectively, confirmed that the analytical sensitivity has been improved by the use of the quantitative POC device.

### 4.2. Clinical Endpoints

In light of these data, the AQT90 βhCG test is suitable for early and advanced pregnancy screening. The same correlation and concordance with the central lab method were obtained in the ED where βhCG is performed by midwives and nurses with limited technical training. POCT βHCG could help in clinical decision making when identifying a positive pregnancy in a premenopausal woman presenting with abdominal pain and/or bleeding or when predicting the viability of the pregnancy in the context of serial βhCG measurements [28]. In both cases, the faster availability of the results could lead to a shorter LOS by helping in effective decision making. All previous studies reported that the introduction of POCT in the ED significantly reduced the delay between the sample acquisition and the test results, but addressing the impact of POCT on flow processing in the ED has given conflicting results [6,7]. In a large prospective cluster-randomized controlled trial involving more than 20,000 patients in the ED, Hausfater et al. observed a significant reduction in the time to result (51 min) in the POCT arm (including one hematology analyzer for blood count; two blood gas analyzers with urea and creatinine; one biochemistry analyzer for liver enzymes, bilirubin, lipase and creatine kinase; and two immunoassay analyzers for troponin T, NT-proBNP, C-reactive protein, procalcitonin, hCG and D-dimer), with no impact on the LOS (−9 min) [29]. In the study conducted by Lewandrowski et al., the implementation of a D-dimer POCT in the ED led to a decrease in the total TAT (from blood draw to the availability of test results) from approximately 2 h to 25 min. It was associated with a significant reduction in the ED LOS from 8.46 to 7.14 h [30]. In a prospective, case-controlled trial of adults presenting to the ED, Singer et al. reported that early POCT at triage reduced the ED care time by approximately 1 h [31]. In accordance with all these studies, we found POCT βhCG implementation to be associated with a significant reduction in the time from blood sampling to test results by 120 min. This shorter response time was further associated with a significant reduction in the LOS by 60 min. As a result, only 50% of the time saved to obtain the result was transformed into a reduced LOS.

In a previous study, we evaluated the impact of POC creatinine testing in ED patients receiving a contrast-enhanced computed tomography scan [32]. Similarly, we found that the TAT required to obtain the creatinine result dropped significantly by 86 min, whereas contrast-induced CT examination was performed about 50 min early with the implementation of ABL800 (*p* = 0.04). Here, again, about 60% of the time saved to obtain the result was transformed into reduced waiting time.

In this study, no time saving was observed for patients treated in less than 40 min, but the number of patients treated in less than 2 h was multiplied by 2.5. Interestingly, the patients in the “LOS > 180 min” group got to be faster, but not the patients in the “120–180 min” group (Figure 3). The magnitude of the effect POCT has on patient care and on efficiency shows a strong dependency on the clinical context. Rapid TATs are the most beneficial if the test results are the primary determining factor holding up patient management decisions [2,3]. The βhCG measurement is just one part of the patient care, and the time required to obtain βhCG results in the central lab is only one of the bottlenecks contributing to the total LOS [2,29]. Factors such as vital signs, patient severity and factors affecting the examination regimen such as day of the week and time of day will have an effect on TAT. The availability of ultrasonography and its interpretation, the need to send other biological parameters to the central lab and the increase in the workload of ED personnel could also explain this frequency of distribution. The workflow procedure can be further optimized by adapting the organization of the ED to the use of the POCT [33,34]. Physicians and midwives knew that they could obtain βhCG results earlier during the POCT period, but work processes remained unchanged during this period of evaluation. Kankaanpää et al. [33] examined the impact of a comprehensive POC test panel, first alone and then with an additional process change in adult ambulatory patients. They demonstrated that a considerable shortening in LOS came from the introduction of POCT (−29 min), and the team’s early assessment process further decreased LOS (−17 min).

### 4.3. Advantages and Disadvantages of the POC

The importance of the POC approach in laboratory monitoring in the acute setting has been recently reviewed by Rajsic et al. [3]. With advances in technology, it has become possible to perform some biomarkers at the bedside with an acceptable level of accuracy [2]. The reduction of TAT, early recognition and the immediate and guided treatment of life-threatening conditions appear to be the most important advantages of POC diagnosis in the intensive care setting [3]. The speed of diagnosis could lead to a potential reduction in treatment time, overall LOS, morbidity and mortality. However, the training and recertification of healthcare workers for POC technology constitute a crucial point to optimize the implementation of POCT in close collaboration with the central lab [3]. The cost effectiveness of POCT is difficult to calculate since too many factors are involved, including reagent costs and the impact on workflow and patient medical care.

### 4.4. Follow-Up and Routine Practice

**Figure 5:**
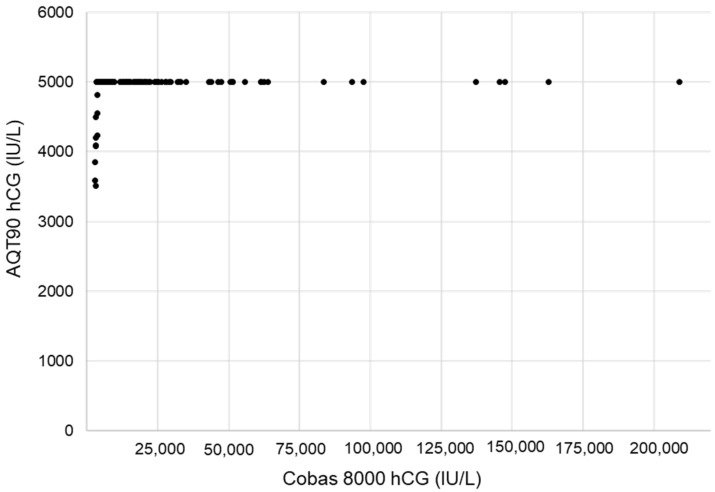
Evaluation of the high-dose hook effect by comparing the hCG results obtained on e602/Cobas 8000 (plasma) and those obtained on AQT90 (whole blood) in the emergency department for samples greater than 3000 IU/L using the Elecsys hCG + β tests (*n* = 180).

Following this study, the βhCG test on AQT has been integrated into the testing strategy of the gynecology ED of our institution since February 2021. Since the permanent installation, 94 operators have been trained on this new system, and 58 are currently active. The precision data generated from our internal quality over the last 15 months point to a long-term CV of 6.3% at the mean value of 26 IU/L (LQC hCG-CHECK level 1) and 5.1% at the mean value of 344 IU/L (LQC hCG-CHECK level 2). High-dose hook effects, which can lead to falsely low results under antigen excess conditions, represent another potential problem in homogeneous immunoassay methods for analytes, with a wide range of concentrations described for βhCG [35,36]. During the study, excess antigen was not determined using spiked samples with varying concentrations of βhCG, but we retrospectively analyzed all the samples run on both systems with βhCG concentrations above 3000 IU/L using the Elecsys hCG + β tests. The samples with hCG concentrations ≤ 210,000 IU/L (*n* = 167) produced the expected result of >5000 IU/L on the AQT90 (Figure 5). One sample with an βhCG value > 1,000,000 IU/L gave the similar result of >5000 IU/L on AQT 90. Our results, in accordance with those previously obtained [15], confirm the manufacturer’s claim of no hook when βhCG concentrations up to 233,000 IU/L are measured.

Our study acknowledges some limitations. Firstly, this is a single-center observational study in a French Gynecology ED, and the results may not be generalizable to other EDs with different organizations and patient characteristics. Secondly, the study was carried out over two different periods, which could have introduced a potential bias of interpretation even if the ED organization remained unchanged between the two periods. The link between POC and LOS could be better validated by a randomized controlled study. Thirdly, our study focused on LOS as the primary outcome, and the time to the decision information presented to an ED physician was not recorded. Fourthly, we did not investigate other factors that might influence the LOS besides POC, such as the availability of ultrasonography and the need to send other biological parameters to the central lab.

In conclusion, the implementation of a rapid quantitative hCG POC method has significantly reduced the time to the results and the LOS in a gynecology ED, with a connection to the middleware ensuring the traceability of the results and allowing for quality management via the central lab.

## Data Availability

The datasets generated during the current study are available from the corresponding author upon reasonable request.
